# *Profundulus kreiseri*, a new species of Profundulidae (Teleostei, Cyprinodontiformes) from northwestern Honduras

**DOI:** 10.3897/zookeys.227.3151

**Published:** 2012-10-05

**Authors:** Wilfredo A. Matamoros, Jacob F. Schaefer, Carmen L. Hernández, Prosanta Chakrabarty

**Affiliations:** 1 Fish Section, Museum of Natural Science, Department of Biological Sciences, Louisiana State University. 119 Foster Hall, Baton Rouge, LA 70803. USA. Phone: 225-578-3079 Fax: 225-578-3075; 2 Department of Biological Sciences, The University of Southern Mississippi, 118 College Dr. Box 5018, Hattiesburg, MS 39406, U.S.A.

**Keywords:** Central America, nuclear Middle America, Cyprinodontiformes, Kreiser’s Killifish, phylogeny, systematics

## Abstract

A new species of *Profundulus*, *Profundulus kreiseri* (Cyprinodontiformes: Profundulidae), is described from the Chamelecón and Ulúa Rivers in the northwestern Honduran highlands. Based on a phylogenetic analysis using cytochrome *b* and the presence of synapomorphic characters (dark humeral spot, a scaled preorbital region and between 32-34 vertebrae), this new species is placed in the subgenus *Profundulus*, which also includes *Profundulus (Profundulus) oaxacae*, *Profundulus (Profundulus) punctatus* and *Profundulus (Profundulus) guatemalensis*. *Profundulus kreiseri* can be distinguished from other members of the subgenus *Profundulus* by having less than half of its caudal fin densely scaled. *Profundulus kreiseri* can further be differentiated from *Profundulus (Profundulus) oaxacae* and *Profundulus (Profundulus) punctatus* by the absence of rows of dark spots on its flanks. The new species can further be differentiated from *Profundulus (Profundulus) guatemalensis* by the presence of fewer caudal- and pectoral-fin rays. The new species is distinguished from congeners of the profundulid subgenus *Tlaloc* (viz., *Profundulus (Tlaloc) hildebrandi*, *Profundulus (Tlaloc) labialis*, *Profundulus (Tlaloc) candalarius* and *Profundulus (Tlaloc) portillorum*) by having a scaled preorbital region and a dark humeral spot. *Profundulus kreiseri* and *Profundulus portillorum* are the only two species of *Profundulus* that are endemic to the region south of the Motagua River drainage in southern Guatemala and northwestern Honduras.

## Introduction

**Figure 1.**
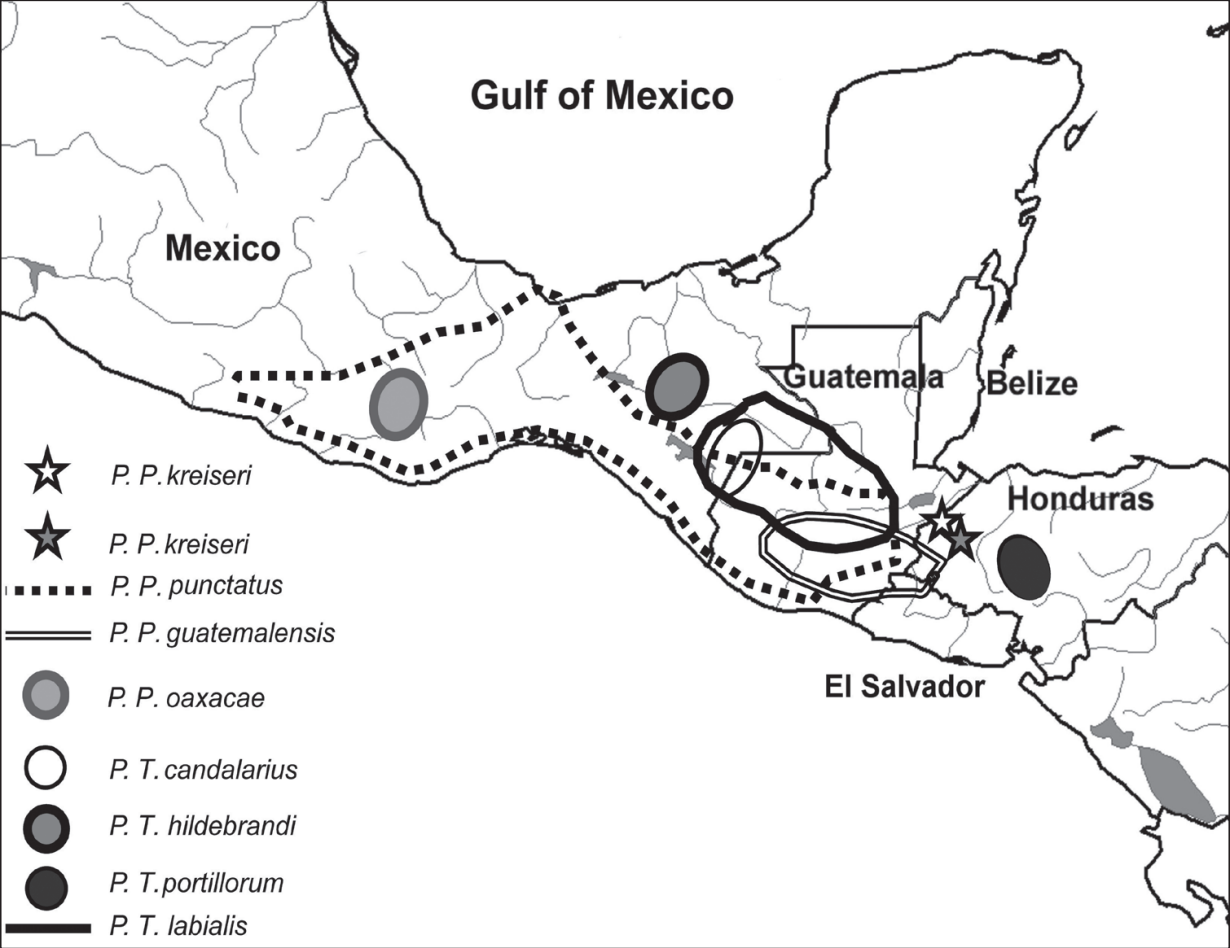
Map of Honduras and neighboring countries showing the distribution of all species of *Profundulus*. The type locality of *Profundulus kreiseri* (15.197667°N, 88.616°W) is represented by a white star, and a second known locality for the species (15.029520°N, 88.508°W) is represented by a dark star.

The genus *Profundulus* Hubbs, 1924, is a depauperate lineage of northern Central American and southern Mexican (Fig. 1) cyprinodontiforms that belong to Profundulidae. The family comprises a single genus, *Profundulus*, which has seven valid species (Matamoros and Schaefer 2010). The northernmost distributed species, *Profundulus oaxacae* (Meek, 1902),is found in the Río Verde basin, in the Mexican Pacific slope in the state of Oaxaca (Miller et al. 2005). The southernmost boundary of the genus is delimited by the distributional range of *Profundulus portillorum* Matamoros and Schaefer, 2010,which is found in the headwaters of the Ulúa and Nacaome River basins in the Atlantic and Pacific slopes of Honduras (Matamoros et al. 2009; Matamoros and Schaefer 2010; Matamoros et al. 2012).

Taxonomic relationships within Profundulidae were inferred by Miller (1955), who erected two subgenera (*Tlaloc* and *Profundulus*). The subgenus *Tlaloc* was diagnosed by having a preorbital region that has either two inconspicuously embedded scales or is naked, small scales on the body, and a high vertebral count. *Tlaloc* includes *Profundulus (Tlaloc) candalarius*, Hubbs 1924, *Profundulus (Tlaloc) hildebrandi*, Miller 1950,* P.T. labialis* (Günther 1866) and the recently described *Profundulus (Tlaloc) portillorum*. The subgenus *Profundulus* is diagnosed by having a preorbital region covered with conspicuous (not deeply embedded) scales, a prominent humeral spot, and at least half (the anterior half) of the caudal fin densely scaled (Miller 1955). The subgenus *Profundulus* includes four species; *Profundulus (Profundulus) punctatus* (Günther, 1866), *Profundulus (Profundulus) guatemalensis* (Günther, 1866), *Profundulus (Profundulus) oaxacae* and the new species described herein.

Among neotropical members of Cyprinodontiformes, profundulids are the least studied. Although some progress has been made over the past two decades in advancing our understanding of the genetics (Doadrio et al. 1999; Valencia-Diaz and Espinoza-Pérez 2011), morphometrics (Gonzales-Diaz et al. 2005) and conservation (Velázquez-Velázquez and Schmitter-Soto 2004; Velázquez-Velázquez et al. 2009) of the family, many aspects of the ecology, life history and general biology of *Profundulus* species remain unknown. Furthermore, most papers that address *Profundulus* are focused on Mexico, the northernmost range of the family, and little is known about the species that occur south of Mexico (e.g., *Profundulus guatemalensis* and *Profundulus portillorum*). Recent ichthyological work in Honduras (Matamoros et al. 2009) and El Salvador (McMahan pers. comm.) has produced new localities and records of undescribed species for the family (e.g. *Profundulus portillorum*). The aim of this paper is to describe a new species of *Profundulus* from the Chamelecón and Ulúa River basins in the Atlantic slope of Honduras.

## Materials and methods

### Morphology

All specimens examined are housed at the University of Southern Mississippi Ichthyology Collection (USM), Louisiana State University Museum of Natural Science (LSUMZ), and University of Michigan Museum of Zoology (UMMZ). Measurements and counts were taken following Miller (1948), including standard length, snout length, head length, predorsal length, anal-fin origin to caudal-peduncle base, anal-fin length, eye diameter, head depth, caudal-peduncle depth, head width and maximum body width. All measurements were made using digital calipers. Counts include midline scales, scales around the caudal peduncle, anal-fin rays, dorsal-fin rays, pectoral-fin rays, and caudal-fin rays.

### Molecular phylogeny

Genomic DNA was extracted from ethanol-preserved fin tissue using a DNeasy Tissue Kit (QIAGEN Inc.). We amplified the mitochondrial cytochrome *b* gene using the GluF and ThrR primers described by Machordom and Doadrio (2001). Initial sequences were generated with these two primers and used to design the following internal primers: ProcytbintF (5’-ACTCGATTCTTYGCCTTCCA-3’) and ProcytbintR (5’-GGGTGAAATGARATTTTGTCG-3’). Subsequent amplifications and sequencing were conducted using the primer pairs GluF-ProcytbintR and ThrR- ProcytbintF. Amplifications were conducted in a total volume of either 25 ml or 50 ml using 50 mM KCl, 10 mM Tris-HCl (pH 8.3), 0.01% gelatin, 200 mM dNTPs, 2 mM MgCl2, 0.5 units of Taq polymerase (New England Biolabs), 0.3 mM of each primer, 20-150 ng of template DNA, and water to the final volume. Cycling conditions consisted of an initial denaturing step of 1 min at 95º C followed by 30 cycles of 1 min at 95º C, 1 min at 50º C and 1 min at 72º C. A final elongation step of 72º C for 3 min completed the reaction. PCR products were cleaned using the ExoSAP-IT system (USB Co.), and then used as the template in a cycle sequencing reaction with an ABI BigDye Terminator cycle sequencing kit (Foster City, CA, USA) using the primers described above. Sequencing reactions were cleaned using sephadex (Princeton Separations, Adelphia, NJ, USA) and then sent to the Iowa State University DNA Sequencing and Synthesis Facility. The sequences were edited and aligned using Sequencher v. 4.10.1 (GeneCodes Co.). Sample information and GenBank accession numbers for ingroup and outgroup species used in the analysis are listed in Table 1.

Sequence divergence (uncorrected p-distance) was estimated in PAUP* (Swofford 2002). The AIC (Akaike’s information criterion) model selection procedure was implemented in jModelTest (Posada 2008) and used to obtain an appropriate substitution model and parameter values for phylogenetic analysis. Phylogenetic relationships were inferred by maximum parsimony (MP) using PAUP* (Swofford 2002) and Bayesian analysis (BA) using MrBayes 3.1.2 (Huelsenbeck and Ronquist 2001). All valid species of *Profundulus* were included in the analysis. Two species from the family Goodeidae, *Allodontichthys hubbsi* Miller & Uyeno, 1980, and *Alloophorus robustus* (Bean, 1982), were used as outgroups in the two phylogenetic analyses: these taxa were selected because Costa (1998) recovered goodeids as the sister group of Profundulidae. For the MP analysis, a heuristic search was performed to find the most parsimonious tree(s). Nonparametric bootstrapping (Felsenstein 1985) was used to measure clade support, with 1000 total pseudoreplicates and TBR branch-swapping with 10 random sequence addition replicates per pseudoreplicate. For BA analysis we performed four simultaneous analyses, each with ten Markov chain Monte Carlo simulations run for 1,000,000 generations, sampling trees every 1000 generations. At the end of the analysis, the average standard deviation of the split frequencies was < 0.01, indicating that the runs had converged. The first 100 trees from each run before reaching equilibrium were discarded as burn-in. The remaining trees were used for reconstruction of a 50% majority-rule consensus tree with posterior probabilities values. One of the paratypes (USM 39024; tissue 08-2921) was sequenced (GenBank accession number JQ254935) and therefore constitutes a “paragenetype cytochrome *b”* following the nomenclature of Chakrabarty (2010).

**Table 1.** GenBank accession numbers and locality information of data used in the molecular analysis. Gu= Guatemala, Me= Mexico, Ho= Honduras.

**Taxa**	**Accession No.**	**Locality**	**Source**
*Profundulus guatemalensis*	AY155568	Gu. Blanco River, San Miguel Duenas	Doadrio and Dominguez 2004
*Profundulus labialis*	AY155567	Gu. Jeronimo River, San Jeronimo	Doadrio and Dominguez 2004
*Profundulus punctatus*	AY155566	Me. Manialtepec Basin, San Juan Lachao	Doadrio and Dominguez 2004
*Profundulus candalarius*	JQ254931	Me. Chiapas, Grijalva River basin	this study
*Profundulus hildebrandi*	JQ254932	Me. Chiapas, San Cristobal de las Casas	this study
*Profundulus kreiseri* n. sp.	JQ254935	Ho. Chamelecón River	this study; USM 39024
*Profundulus kreiseri* n. sp.	JQ254934	Ho. Ulúa River	this study; USM 39028
*Profundulus oaxacae*	JQ254933	Me. Oaxaca, Verde River basin	this study
*Profundulus portillorum*	JQ254929	Ho. Nacaome River	this study; USM 31628
*Profundulus portillorum*	JQ254930	Ho. Ulúa River	this study; USM 31597
*Allodontichthys hubbssi*	AF510835	Me. Jalisco, El Trampolin	Doadrio and Dominguez 2004
*Alloophorus robustus*	AF510809	Me. Michoacán, Uruapan	Doadrio and Dominguez 2004

### Usage of “genetypes” nomenclature

The “genetypes” nomenclature was proposed by Chakrabarty (2010) to flag sequences from type specimens. Many genetypes can be created from a single specimen and these may be a single gene region or an entire genome; for instance “paragenetype COI” and “paragenetype ND2” could be added from USM 39024 at a later date, as could genetypes from other type specimens of this species (e.g., paratypes, holotype) from which DNA can be extracted. This nomenclature is simply a flag to alert molecular biologists and taxonomists that sequences are available from type specimens.

The genetype terminology is not used here in a strict nomenclatural sense, as it is not formally accepted by the International Code for Zoological Nomenclature. However, we consider this terminology useful and expect increased application in the future. The present paper is one of the first uses of the genetype terminology.

## Results

### Systematic Account

#### Profundulus kreiseri sp. n.

urn:lsid:zoobank.org:act:97D8525F-9BDF-48EF-A7C5-21AD7917558B

http://species-id.net/wiki/Profundulus_kreiseri

Figure 2

Profundulus sp. 2 Matamoros et al. 2009
Profundulus sp. 2 Santa Barbara Matamoros et al. 2012

##### Type material.

**Figure 2.**
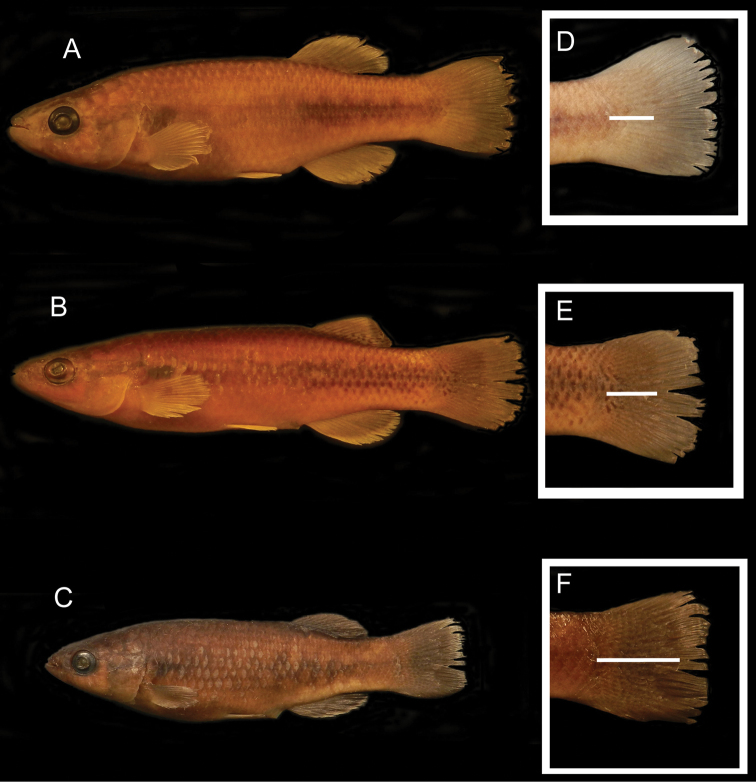
Photographs of preserved specimens of **A** *Profundulus kreiseri* sp. n., USM 39022, holotype, 53.2 mm SL, Río Chamelecón, Honduras **B** *Profundulus punctatus*, UMMZ 194154, 47.0 mm SL, Rio Nahualete, Guatemala; and **C** *Profundulus guatemalensis*, UMMZ 190542, 43.7 mm SL, Río Maria Linda, Guatemala. The diagnostic characters of each species (**A–C**) are shown in **D–F** the white bar shows the extent of squamation on the caudal fin **D** *Profundulus kreiseri* sp. n., USM 39022, holotype (note that scales cover less than half of the caudal fin, and there are no rows of dark spots on the caudal peduncle and caudal-fin base) **E** *Profundulus punctatus*, UMMZ 194154 (note that the entire anterior half of the caudal fin is covered with scales, and rows of dark dots occur on the caudal peduncle and caudal-fin base; and **F** *Profundulus guatemalensis*, UMMZ 190542 (note that scales extend beyond the midpoint of the caudal fin, and there are no rows of dark spots on the posterior portion of the caudal peduncle and caudal-fin base).

Holotype: USM 39022, field number WAM09-28, Honduras, Department of Santa Barbara, Municipality of Macuelizo. Drainage: Chamelecón, System: Chamelecón. Locality: small creek that drains to the Chamelecón River, near the Chamelecón Hydroelectric Dam 15.197667°N, 88.616°W; Collectors: W.A. Matamoros, M. Medina and J.C. Carrasco, 3 July 2009 (Fig. 2).

Paratypes: LSUMZ 14851 (n=9), field number WAM08-141, Honduras, Department of Santa Barbara, Drainage: Ulúa, small creek that drains into the main river, 15.029520°N, 88.508°W, Collectors: W.A. Matamoros, F. Elvir and H. Vega, 7 August 2008; LSUMZ 14852 (n=4), same data as the holotype; USM 39024 (n=9), same data as the holotype; USM 39025 (n=7), same data as LSUMZ 14851; USM 39026 (n=5), same data as LSUMZ 14851.

##### Diagnosis.

*Profundulus kreiseri* is a new member of the subgenus *Profundulus* and shares with other members of that subgenus (viz., *Profundulus (Profundulus) punctatus*, *Profundulus (Profundulus) guatemalensis* and *Profundulus (Profundulus) oaxacae*) the following set of characters: dark humeral spot, a scaled preorbital region and between 32-34 vertebrae. It differs from all members of the subgenusby having less than half of its caudal fin densely covered with scales. It can further be distinguished from *Profundulus (Profundulus) oaxacae* and *Profundulus (Profundulus) punctatus* by the absence of conspicuous brown spots on the sides of the body; from *Profundulus (Profundulus) guatemalensis* by having fewer pectoral-fin rays (17-20 in *Profundulus (Profundulus) guatemalensis* versus 13-16 in *Profundulus kreiseri*) and caudal-fin rays (19-23 in *Profundulus (Profundulus) guatemalensis* versus 13-18 in *Profundulus (Profundulus) kreiseri*); and from *Profundulus (Profundulus) guatemalensis* and *Profundulus (Profundulus) punctatus* by having a golden blotch that covers most of the operculum and reaches the base of the pectoral fin. *Profundulus (Profundulus) kreiseri* is distinguishable from all members of the subgenus *Tlaloc* (viz., *Profundulus (Tlaloc) candalarius*, *Profundulus (Tlaloc) hildebrandi*, *Profundulus (Tlaloc) labialis*, *Profundulus (Tlaloc) portillorum*) by having a dark humeral spot and a scaled preorbital. *Profundulus (Profundulus) kreiseri* can further be differentiated from *Profundulus (Tlaloc) candalarius*, *Profundulus (Tlaloc) hildebrandi* and *Profundulus (Tlaloc) labialis* by having between 32-34 vertebrae (versus 35-39).

##### Description.

Morphometric and meristic data for type material are summarized in Table 2. The largest specimen is 81.2 mm SL. The body is elongate with the dorsal and ventral profiles nearly symmetrical. The narrowest point on the body is the tip of the snout, with the body expanding gradually dorsally and ventrally to the deepest point slightly anterior to the verticals through the dorsal- and anal-fin origins. The vertical through the origin of the dorsal fin is slightly anterior to the origin of the anal fin. The body depth narrows in the region of these two unpaired fins, and the dorsal and ventral body margins are straight and parallel on the caudal peduncle before diverging out slightly at the origin of the caudal fin.

The head (including cheek, infraorbital and preorbital regions) is covered with scales that are deeply embedded in the skin. The mouth is terminal, the lower jaw protruding slightly beyond the upper. The posterior portion of the maxilla extends ventrally to a vertical through the anterior region of the orbit.

The number of dorsal-fin rays ranges from 10-12 (holotype=11). The number of pectoral-fin rays ranges from 13-16 (holotype=14). The posterior edge of the pectoral fin does not reach the pelvic-fin origin. The number of anal-fin rays ranges from 9-14 (holotype=12; mode=12). The caudal fin is rounded, the number of fin rays ranging from 13-18 (holotype=17). All specimens examined have six pelvic-fin rays. The number of scales along the midline of the body ranges from 33-34 (holotype=33). The number of scales around the caudal peduncle ranges from 9-10 (holotype=10). The number of vertebrae ranges from 31-33 (holotype=32).

##### Live coloration.

In life this species is brown, with a golden-yellow blotch that covers most of the operculum and reaches the base of the pectoral fin. An inconspicuous dark stripe is present along the midline of the body starting at a vertical between the dorsal- and anal-fin origins and terminating at the origin of the caudal fin.

##### Preserved coloration.

Thebody is a uniform dusky brown with a prominent dark humeral spot posterior to the upper insertion of the pectoral fin. A dark stripe is present along the midbody; this stripe is more conspicuous in preservation than in life. The distal margins of the unpaired fins are opaque, but the basal ¾ of the fins are covered with scattered melanophores.

##### Distribution.

**Figure 3.**
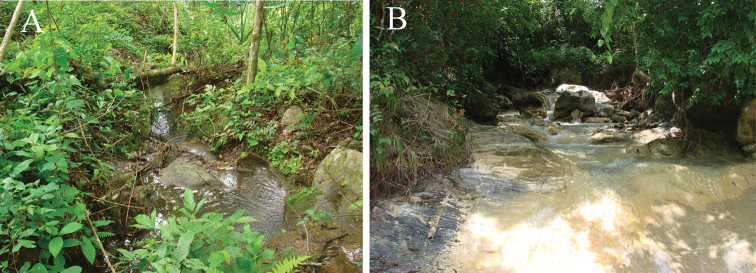
Photographs of the habitat of *Profundulus kreiseri*. **A** Type locality of *Profundulus kreiseri*, 15.197667°N, 88.616°W. A small creek that drains directly into the Chamelecón River, near the new hydroelectric dam. Department of Santa Barbara, Municipality of Macuelizo **B** Small creek that drains into the main river. Department of Santa Barbara, 15.029520°N, 88.508°W.

*Profundulus kreiseri* is only known from the middle reaches of the Chamelecón and Ulúa rivers in Honduras (Figs. 1, 3).

**Ecological notes.** The only known localities of *Profundulus kreiseri* are both characterized as small tributaries ranging in width from 0.8 to 4 meters with stones (from pebbles to boulders) as the dominant substrate. The canopy cover of both localities is estimated to be 70–80%. Both creeks feature a variety of run, pool, riffle, rapid and small waterfall habitats (Fig. 3).

##### Conservation.

The limited range of this species makes it vulnerable to extinction via habitat loss. The creation of a new hydroelectric dam on theChamelecón River will likely drastically impact populations of this new species.

##### Etymology.

The specific epithet is in honor of Dr. Brian R. Kreiser, the doctoral advisor and friend of the first author.

Suggested English name: Kreiser’s Killifish

Suggested Spanish name: El Escamudo de Kreiser

**Table 2.** Morphological data and counts from the holotype and 33 paratypes of the new species *Profundulus kreiseri*. Measurements are presented as % of standard length (%SL) or % of head length (%HL). SD=standard deviation.

**Morphological data**	**Holotype**	**Range**	**Mean**	**SD**
Standard length	53.2	46.1–81.2	60.3	9.7
Snout length (%HL)	30.1	20.4–36.1	28.9	3.6
Head length (%SL)	26.0	24.0–28.8	25.8	1.2
Predorsal length (%SL)	70.9	20.4–74.9	68.9	2.2
Anal-fin origin to caudal-peduncle base (%SL)	32.0	29.6–35.7	31.9	1.7
Anal-fin length (% SL)	32.0	28.5–34.9	31.9	1.8
Eye diameter (%HL)	23.0	20.4–29.5	23.4	2.1
Head depth (%HL)	75.6	42.8–78.1	69.4	6.6
Maximum body depth (%SL)	24.9	18.1–25.7	23.2	2.0
Caudal-peduncle depth (%SL)	14.0	12.0–15.5	13.8	0.8
Head width (%HL)	66.9	36.2–68.1	61.2	6.6
Maximum body width (%SL)	17.4	10.2–19.5	16.0	1.8

### Molecular analysis

The phylogenetic analysis included sequences from 12 taxa (10 in the ingroup and 2 outgroups). Novel sequences were deposited in GenBank under accession numbers JQ254929-JQ254935 (Table 1). A total of 990 bp of cytochrome *b* were analyzed, of which 280 sites (28.3%) were parsimony informative. The GTR + I + G model was selected as the best fit for the dataset by jModelTest using the AIC. The optimized parameters were: A=0.2692, C=0.2640, G=0.2334, T= 0.3335, Rmat= (8.4428, 56.7004, 8.4428, 1.0000, 56.7004, 1.0000), the gamma distribution was 1.4610, and the proportion of invariable sites was 0.4970.

**Figure 4.**
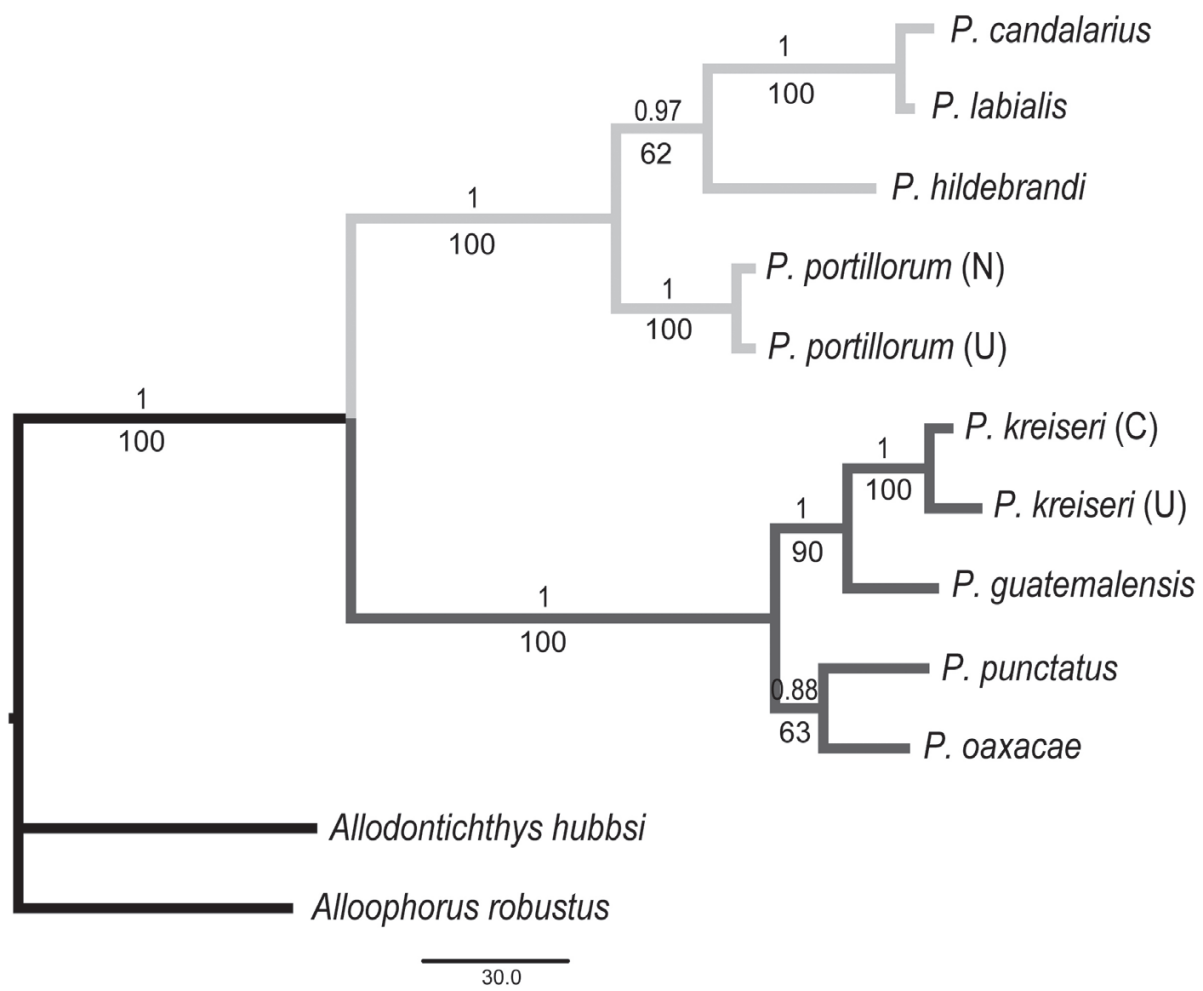
Bayesianand maximum parsimony trees based on cytochrome *b* sequences. Number above each node represents Bayesian posterior probabilities. Number below each node represents nonparametric bootstrap support. Subgenus membership is shown with either light gray branches (*Tlaloc*) or dark gray branches (*Profundulus*). C = Chamelecón River, U = Ulúa River, N = Nacaome River.

The same tree topology was recovered by MP (a single tree, length: 585, CI: 0.735; RI: 0.796) and BA (Fig. 4). Two monophyletic groups corresponding to the two subgenera were recovered. Node support was generally high throughout the tree (Fig. 4). The new species was recovered as the sister group of *Profundulus guatemalensis* with high bootstrap and posterior probability values. P- distances between the members of the two subgenera ranged from 17.8 to 19.3%. Within the subgenus *Tlaloc*, sequence divergence ranged from 1 to 9.2%. The shortest genetic distance was found between *Profundulus labialis* and *Profundulus candalarius* (1%). Sequence divergence between the new species (two specimens) and each of the three other species in the subgenus *Profundulus* ranged from 4.1 to 6.5% (Table 3).

**Table 3.** Uncorrected pairwise genetic divergence based on cytochrome *b* sequences. C= chamelecón river, u= ulúa river, n= nacaome river.

	**1**	**2**	**3**	**4**	**5**	**6**	**7**	**8**	**9**
1. *Profundulus (Profundulus) kreiseri* (C)	0								
2. *Profundulus (Profundulus) kreiseri* (U)	0.016	0							
3. *Profundulus (Profundulus) guatemalensis*	0.041	0.047	0						
4. *Profundulus (Profundulus) punctatus*	0.062	0.064	0.064	0					
5. *Profundulus (Profundulus) oaxacae*	0.063	0.065	0.063	0.040	0				
6. *Profundulus (Tlaloc) portillorum* (U)	0.185	0.184	0.182	0.181	0.178	0			
7. *Profundulus (Tlaloc) portillorum* (N)	0.187	0.186	0.183	0.180	0.178	0.008	0		
8. *Profundulus (Tlaloc) hildebrandi*	0.191	0.191	0.186	0.183	0.184	0.080	0.080	0	
9. *Profundulus (Tlaloc) labialis*	0.192	0.193	0.183	0.187	0.186	0.087	0.087	0.078	0
10. *Profundulus (Tlaloc) candalarius*	0.192	0.193	0.183	0.183	0.187	0.090	0.092	0.083	0.010

## Discussion

*Profundulus portillorum* has the southernmost range of any species in the family; it is found in the Ulúa River in the Atlantic slope of Honduras and the Nacaome River in the Pacific slope of Honduras (Matamoros and Schaefer 2010). The Chamelecón River locality reported for *Profundulus kreiseri* represents a new drainage for the family. Most species of *Profundulus* have restricted distributional ranges (found only among a few adjacent river systems) and only one species has a widespread distributional range, *Profundulus guatemalensis*. This species is the only *Profundulus* to cross the Motagua River Fault into nuclear Middle America, a region that extends from southern Guatemala to northern Nicaragua (Matamoros et al. 2012). The Motagua River Fault may be a biogeographic boundary for *Profundulus*. *Profundulus punctatus* and *Profundulus labialis* also reach the Motagua River, but are not found south of it (Miller 1955; Miller et al. 2005; Matamoros and Schaefer 2010) and *Profundulus portillorum* and *Profundulus kreiseri* are restricted to nuclear Middle America but are not found north of the Motagua River. The combination of the tree topologies, node support, uncorrected pairwise divergences values and morphological comparisons between the new species and its congeners fully support the distinctiveness of *Profundulus kreiseri*.

*Profundulus kreiseri* has higher intraspecific genetic variation (0.016) than is found between some species (e.g., 0.010 in *Profundulus labialis* versus *Profundulus candalarius*). The low sequence divergence found between *Profundulus labialis* and *Profundulus candalarius* may be a reflection of a recent divergence. These two taxa are currently allopatric and can be differentiated based on morphological characters (e.g., fin ray counts, body depth). However, additional work may be needed to verify the taxonomic status of these taxa given the results from our molecular analysis. Intraspecific variation in *Profundulus kreiseri* is also twice as high as what was recovered for the only other species for which multiple individuals were sampled in our study, *Profundulus portillorum* (0.008). This variation in *Profundulus kreiseri* may reflect a relatively long period of isolation between the populations in the Chamelecón and Ulùa rivers.

Our molecular phylogenetic analysis recovered two distinct clades corresponding to the nominal subgenera and placed *Profundulus kreiseri* within the subgenus *Profundulus* as the sister taxon of *Profundulus guatemalensis*.The caudal-fin scales of *Profundulus kreiseri* do not extend beyond the anterior half of the fin (Fig. 2D), whereas they cover half or more of the caudal fin in other members of the subgenus *Profundulus* (e.g., *Profundulus punctatus* and *Profundulus guatemalensis*—Fig. 2E, F). Because the presence of squamation on at least half of the caudal fin is a defining character of the subgenus (Miller 1955), our recognition of *Profundulus kreiseri* as a member of the subgenus *Profundulus* is based on the molecular data and the presence in *Profundulus kreiseri* and other members of the subgenus of a dark humeral spot and scaled preorbital region. The new species exhibits none of the diagnostic features of the subgenus *Tlaloc*. In addition to the caudal squamation, species status of *Profundulus (Profundulus) kreiseri* is warranted based on its pigmentation pattern and numbers of pectoral- and caudal-fin rays. A dichotomous key to all of the Central American species of *Profundulus* is presented below.

### Key to species of *Profundulus* from Central American

**Table:** 

1a	Preorbital region usually naked, frequently with one or two isolated inconspicuous embedded scales; humeral spot absent	2
1b	Preorbital region with at least three to four conspicuous (not deeply embedded under the skin) scales; humeral spot present	4
2a	Lower jaw protruding beyond upper jaw	*Profundulus labialis*
2b	Lower and upper jaws same length	3
3a	Pectoral-fin rays 16 – 19. Endemic to the Grijalva-Usumacinta River basin	*Profundulus candalarius*
3b	Pectoral-fin rays 13 – 16. Endemic to Honduras	*Profundulus portillorum*
4a	Body of adults with conspicuous brown spots (distinctly within the scales) in longitudinal rows on the midline from the midpoint of the body to the caudal fin; dorsal surface of head concave to nearly flat	*Profundulus punctatus*
4b	Body of young and adults lacking distinct brown spots (as described in 4a); dorsal surface of head convex (rounded)	5
5a	Scales on the caudal fin covering at least the anterior half or more of the fin. Caudal-fin rays 19-23, very rarely 18 or 24; pectoral-fin rays 17-20, very rarely 16 or 21	*Profundulus guatemalensis*
5b	Scales on the caudal-fin covering less than half of the fin. Caudal-fin rays 13-18, pectoral-fin rays 13–16.	*Profundulus kreiseri* sp. n.

#### Comparative material.

*Profundulus candalarius*: UMMZ 1767317, n = 10, 23–44 mm SL, Guatemala,Huehuetenango, tributary of Río Lagartero in Ciénega Lagartero; UMMZ 2108276, n = 10, 41–66 mm standard length, LS,Mexico, Chiapas, Arroyo tributary to Río Comitan, 11.5 km east of Highway 190 onthe road to Montebello National Park. *Profundulus guatemalensis*: UMMZ 1905421, n = 15, 43–62 mm SL, Guatemala, Escuintla, Río Marinala at Finca Peña Blanca 11 km north-east Escuintla; UMMZ 2191352, n = 4, 37–49 mm SL, Honduras, Río Copan bridge crossing on Ca 11, 26.4 km east of Copan.

*Profundulus punctatus*: UMMZ 1847303, n = 10, 42–51 mm SL, Mexico, Chiapas, Stream at Piedra Parada, 12.1 km west-north-west of Ocozocoautla; UMMZ 1941544, n = 4, 54–70 mm SL, Escuintla, Guatemala, Río Siguacan at Ca Highway 2, km 120, 8 km east of Río Bravo; UMMZ 1941605, n = 5, 55–66 mm SL, Escuintla, Guatemala, Río Aguna at CA 2, km 98, 15 km west-north-west.

*Profundulus hildebrandi* : UMMZ 1576348, 15, 46–103 mm SL, Mexico, Chiapas, Laguna de Maria Eugenia, San Cristobal de las Casas; UMMZ 1839149, n = 5, 33–47 mm SL, Mexico, Chiapas, Irrigation ditch of Laguna Maria Eugenia at Highway. 190, 2.4 km southwest of San Cristobal de las Casas. *Profundulus labialis*: UMMZ 16669811, n = 5, 50–71 mm SL, Guatemala, Rio Carchela c. 29·0 km north of Salama on road to Coban; UMMZ 17672610, n = 15, 43–64 mm SL, Guatemala, Huehuetenango, stream through pasture southwest of Jacaltenango.

*Profundulus portillorum*: USM 31597, n = 31, field number WAM07-03, Honduras, department of Comayagua, municipality of Potrerillos, in the town of Siguatepeque. Drainage: Ulúa. System: Ulúa: Río Calam. Quebrada de Potrerillos at Barrio San José, 14.53000°N, 87.84000°W, Collectors: Matamoros W.A. and H. Portillo; USM 31628, n = 21, field number WAM07-39, Honduras, department of Francisco Morazan, municipality of Lepaterique, Basin: Pacific. Drainage: Nacaome, System: Nacaome, Quebrada El Sapo, near the community of Lepaterique, 100 meters away from Catholic Church, 14.064275°N, 87.466850°W, Collectors: Matamoros W.A., A. Sanchez, E. Lopez and J. Hernandez.

*Profundulus kreiseri*: USM 39028, 9, field number WAM08-141, Honduras, department of Santa Barbara, Drainage: Ulúa, System: Ulúa, small creek that drains in to main river. 15.029520°N, 88.508°W. Collectors: W.A. Matamoros, F. Elvir and H. Vega; USM 39027, n = 3, same data as USM 39028.

## Supplementary Material

XML Treatment for Profundulus kreiseri
